# Lumbosacral transitional vertebrae alter the distribution of lumbar mobility–Preliminary results of a radiographic evaluation

**DOI:** 10.1371/journal.pone.0274581

**Published:** 2022-09-29

**Authors:** Luis Becker, Lukas Schönnagel, Tim Victor Mihalache, Henryk Haffer, Friederike Schömig, Hendrik Schmidt, Matthias Pumberger

**Affiliations:** 1 Center for Musculoskeletal Surgery, Charité –University Medicine, Berlin, Germany; 2 Berlin Institute of Health, Julius Wolff Institute for Biomechanics and Musculoskeletal Regeneration, Charité –University Medicine, Berlin, Germany; Rush University Medical Center, UNITED STATES

## Abstract

**Background:**

Lumbo-sacral transitional vertebrae (LSTV) are one of the most common congenital variances of the spine. They are associated with an increased frequency of degeneration in the cranial adjacent segment. Hypermobility and concomitant increased loads are discussed as a possible reason for segmental degeneration. We therefore examined the lumbar and segmental motion distribution in patients with LSTV with flexion-extension radiographs.

**Methods:**

A retrospective study of 51 patients with osteochondrosis L5/S1 with flexion and extension radiographs was performed. Of these, 17 patients had LSTV and were matched 1:1 for age and sex with patients without LSTV out of the collective of the remaining 34 patients. The lumbar and segmental range of motion (RoM) by segmental lordosis angle and the segmental wedge angle were determined. Normal distribution of parameters was observed by Kolmogorov-Smirnov-test. Parametric data were compared by paired T-test. Non-parametric data were compared by Wilcoxon-rank-sum-test. Correlations were observed using Spearman’s Rank correlation coefficient. A p-value <0.05 was stated as statistically significant.

**Results:**

Patients with LSTV had mean age of 52.2±10.9, control group of 48.9±10.3. Both groups included 7 females and 10 males. Patients with LSTV presented with reduced RoM of the lumbar spine (LSTV 37.3°±19.2°, control 52.1°±20.5°, p = 0.065), however effects were statistically insignificant. LSTV significantly decreased segmental RoM in the transitional segment (LSTV 1.8°±2.7°, control 6.7°±6.0°, p = 0.003). Lumbar motion distribution differed significantly; while RoM was decreased in the transitional segment, (LSTV 5.7%, control 16.2%, p = 0.002), the distribution of lumbar motion to the cranial adjacent segment was increased (LSTV 30.7%, control 21.6%, p = 0.007).

**Conclusion:**

Patients with LSTV show a reduced RoM in the transitional segment and a significantly increased motion distribution to the cranial adjacent segment in flexion-extension radiographs. The increased proportion of mobility in the cranial adjacent segment possibly explain the higher rates of degeneration within the segment.

## Introduction

Lumbo-sacral transitional vertebrae (LSTV) are one of the most common congenital anomalies of the spine with a reported prevalence of 9.9–29% in large scale studies of general population [1–4]. LSTV are classified according to Castellvi, assessing the enlargement and fusion of the processus transversus with the sacral ala [5]. The association between LSTV and back pain was first reported by Bertolotti probably caused by a pseudarthrosis between the widened processus transversus and the sacral bone and a consecutive irritation at the contact area [6]. In addition, extraspinal nerve compression through the widened processus transversus has been described [7]. An increased degeneration of the cranially adjacent segment to the LSTV is also suspected to be a reason for back pain in patients with LSTV. There is a consensus in the literature regarding increased disc degeneration [8–12] and a higher incidence of facet joint degeneration and neuroforaminal stenosis in the cranial segment adjacent to LSTV [11, 12] may be caused by an altered load transfer [6, 10, 13]. A possible instability of the vertebral segment above the transitional vertebra caused by a weak iliolumbar ligament could lead to subsequent disc degeneration. A reduced mobility between the transitional vertebra and the sacrum could be preserved by the formation of either an articulation or by bony union between the vertebra and the sacrum through its transverse process [10]. Furthermore, a reduced mobility of the transitional segment is discussed and attributed to an increased osseous connectivity of the transversal process of the LSTV with the sacral ala with a compensatory hypermobility of the cranially adjacent segment [6, 13]. The results are mainly based on in vitro analyses, whose used protocols have never been validated against in vivo kinematic data. Whereas in vitro studies before and after segmental fusion presume the same overall mobility of the lumbar spine, in vivo studies show that patients after spinal fusion rather decreased the motion of the whole lumbar spine, and thus protect the adjacent segment to fusion from increased mobility [14, 15].

To date, there is a lack of motion analyses of patients with LSTV describing the mobility in the LSTV segment as well as the cranially adjacent segments in vivo. Therefore, the aim of this study was to investigate the mobility of the lumbar spine and segmental motion distribution of patients with LSTV in flexion-extension radiographs.

## Methods

### Patient cohort

This study was performed as retrospective matched-pair analysis. The institutional ethics committee of the Charité University Berlin (EA4/155/21) approved the study. Informed consent from the patients was waived due to the retrospective study design according to ethics committee approval. The study was carried out according to the declaration of Helsinki. We included patients who were evaluated as part of the preoperative preparation for anterior lumbar interbody fusion from 01/2016 to 05/2021 with flexion and extension imaging of the lumbar spine as a consecutive case series due to osteochondrosis L5/S1. We included patients older than 18 years of age. Exclusion criteria were scoliosis with a Cobb angle >20°, spondylolisthesis, previous spondylodesis, suspected spondylodiscitis, lack of preoperative full spine radiographs in the standing position or flexion and extension radiographs. Fifty-one patients were finally included, 17 had LSTV (33.3%). These were matched 1:1 for age and sex with a control group without LSTV out of the remaining 34 patients.

### Classification

LSTV were classified according to Castellvi by both an orthopedic resident surgeon with three years of experience as well as a spine surgeon with eleven years of experience. Classification according to Castellvi is given in Table 1. Phönix-PACS software (Phönix-PACS GmbH, Freiburg im Breisgau, Germany) was used for measurements. The number of lumbar vertebral bodies was classified by counting caudally from C1 in whole-spine images. For the cervical spine, seven vertebrae were assumed, and twelve for the thoracic spine. L1 was defined as the 20th vertebra. We assumed six lumbar vertebrae if we counted 25 vertebrae with an at least rudimentary disc in between. Therefore, in cases of six lumbar vertebrae the transitional segment was level L6/S1, in all other cases the transitional segment was L5/S1. The cranially adjacent segment was consequently in patients with six lumbar vertebrae segment L5/6, in all other patients L4/5.

**Table 1:** Radiographic classification for lumbosacral transitional vertebrae (LSTV) according to Castellvi [5].

Castellvi Type	Definition
Type I: dysplastic transverse process	Uni- (A) or bilateral (B) transverse process with a height >19 mm
Type II: incomplete lumbarization/ sacralization	Uni- (A) or bilateral (B) pseudarthrosis of the enlarged transverse process with the sacral ala
Type III: complete lumbarization/ sacralization	Uni- (A) or bilateral (B) bony fusion of the enlarged transverse process with the sacral ala
Type IV: mixed	Unilateral pseudarthrosis and contralateral bony fusion of the enlarged transversal process with the sacral ala

### Image acquisition and measurements

Full spine radiographs were obtained using biplanar low dose stereoradiography (EOS, Paris, France) from lateral and anterior posterior in standing position. Functional images were obtained with lateral X-ray trajectory as ventral flexion and dorsal extension. Ventral flexion and dorsal extension were acquired in standing position and the patient was instructed to fully flex/extend the spine, while a bar limited tilting of the pelvis.

**Fig 1:**
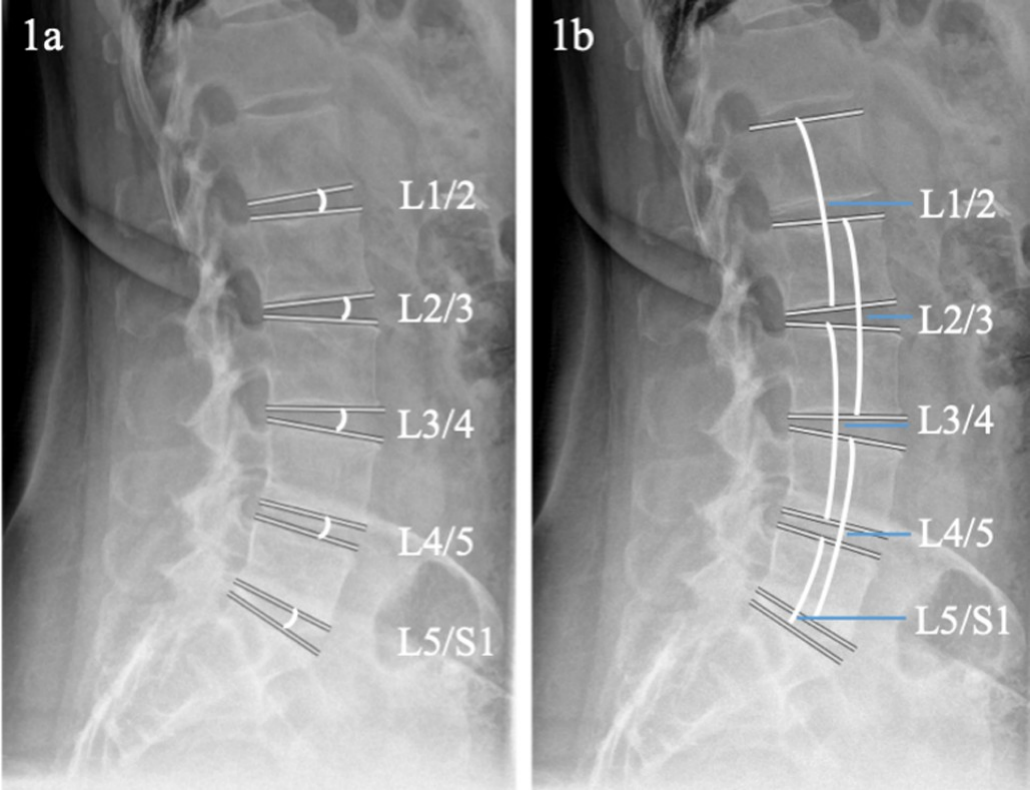
Measurement of segmental wedge angle and segmental lordosis angle. a. shows the measurement of the segmental wedge angle of the lumbar segments. b. depicts the measurement of the segmental lordosis angle.

All parameters were measured by two of the authors. Lumbar lordosis (LL) was measured as the angle of the L1 upper endplate to the S1 upper endplate. Segmental wedge angles were measured between the upper endplate of the lower vertebral body and the lower endplate of the upper vertebral body as depicted in Fig 1A. Segmental lordosis angles were measured between the upper endplate of the upper vertebral body and the lower endplate of the lower vertebral body as shown in Fig 1B. For the lowest segment, segmental lordosis angle was measured between S1 upper endplate and upper endplate L5 or L6. Measurements were performed independently by two orthopedic resident surgeons with three years and two years of experience, after being trained by a spine surgeon with eleven years of experience. The range of motion was calculated as the sum of kyphosis in flexion and increased lordosis in extension. The segmental contribution to the total lumbar motion was determined by the percentages of the total sum of the range of motion of all lumbar segments.

**Fig 2:**
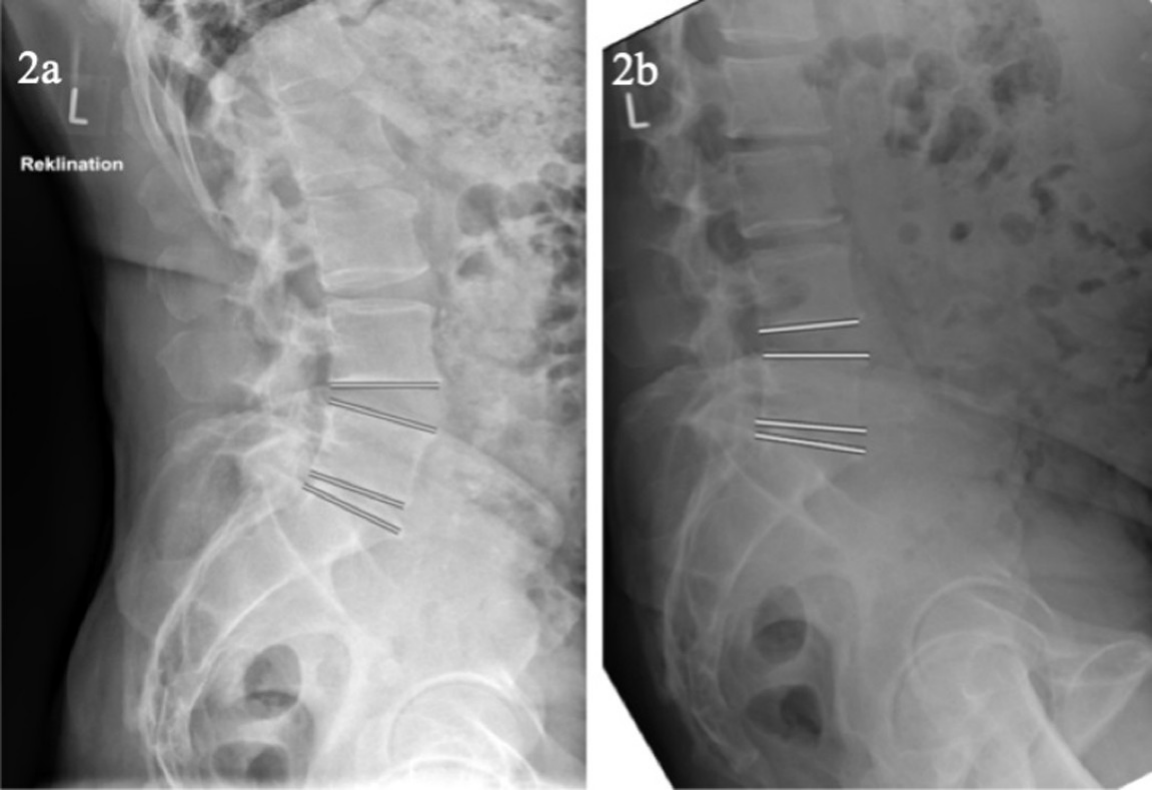
Example of a patient with reduced mobility in transitional segment L5/S1 and compensatory enhanced mobility in the cranial adjacent segment. a. depicts the dorsal trunk extension with increased segmental and lumbar lordosis, which results mostly of the cranially adjacent segment to the transitional segment L5/S1 in this participant. b. depicts the ventral trunk flexion with reduced segmental and lumbar lordosis.

In Fig 2 an example of flexion-extension radiographs of a participant with Castellvi IIb and reduced mobility in transitional segment and enhanced mobility in the cranial adjacent segment is given.

### Statistics

Statistical analyses were performed using SPSS Version 27 (IBM Corporation, New York, USA). The Kolmogorov-Smirnov test was used to test the data for normal distribution. For statistical analysis of parametric paired data, the paired T test was used. For nonparametric paired data, the Wilcoxon rank sum test was used. For testing correlations, Pearson’s correlation coefficient was used for parametric data. Spearman’s correlation coefficient was used for nonparametric data. The significance level was set at p < 0.05 for all tests. Interrater reliability between the two raters for quantitative data was tested using intraclass coefficient, for categorial data by Cohens kappa.

## Results

### Demographics

The cohort of patients with LSTV had a mean age of 52.2 years (range 29–74 years), matched control group 48.9 years (range 36–71 years). Both groups included seven females and ten males. Four patients of the 17 patients with LSTV had six lumbar vertebrae (6LV). One of the 17 patients with LSTV had four free lumbar vertebrae (4LV). Four out of the 17 patients with LSTV had a transitional vertebra Castellvi I, nine had an LSTV Castellvi II, three had an LSTV Castellvi III and one patient had an LSTV Castellvi IV. An excellent interrater reliability for the grading of LSTV according to Castellvi with kappa of 0.924 (p<0.001) was observed. A high degree of reliability was found for the measurements of lumbar lordosis, segmental wedge angles and segmental lordosis between the two observers. The resulting interclass correlation coefficient was 0.971 with a 95% confidence interval from 0.968 to 0.975 (p<0.001).

### Lumbar motion

Patients with LSTV and the control group did not differ significantly in lumbar lordosis in the upright standing position (p = 0.875). No significant differences were found between patients with LSTV and the control group for lumbar RoM (p = 0.065) if it was evaluated as Cobb angle from L1 upper endplate to S1-endplate. Also when looking separately to ventral flexion (p = 0.083) and dorsal extension (p = 0.426) no differences could be detected for measurement from L1 upper endplate to S1 upper endplate. Considering the motion resulting from disc deformation measured by summed segmental wedge angles, patients with LSTV presented reduced lumbar RoM (p = 0.022) resulting from reduced lumbar flexion (p = 0.017). However, lumbar extension was not reduced compared to the control group (p = 0.535). Lumbar movement is presented in Table 2.

**Table 2:** Lumbar lordosis and range of motion in patients with LSTV and control group.

	S1-endplate to L1 upper endplate			Segmental wedge angle		
	LSTV Mean (SD)	Control Mean (SD)	p-value	LSTV Mean (SD)	Control Mean (SD)	p-value
Lumbar lordosis [°]	43.7 (±7.2)	43.8 (±11.6)	0.875	43.7 (±7.2)	43.8 (±11.6)	0.875
RoM flexion [°]	32.0 (±16.5)	44.1 (±19.2)	0.083	20.5 (±9.4)	30.7 (±13.5)	**0.017**
RoM extension [°]	5.3 (±8.2)	8.0 (±8.6)	0.426	13.4 (±5.0)	15.0 (±6.8)	0.535
Lumbar RoM [°]	37.3 (±19.2)	52.1 (±20.5)	0.065	33.9 (±11.0)	45.8 (±14.8)	**0.022**

In Table 2 the mean and standard deviation of lumbar lordosis in upright standing position, ventral flexion, dorsal extension is presented of patients with LSTV and control group. The lumbar range of motion was calculated as the sum of extension and flexion. The lumbar lordosis, flexion and extension of the lumbar wedge angles was calculated as the sum of movement in each lumbar disc. SD = standard deviation, RoM = range of motion. Level of significance was set at 0.05.

### Segmental mobility

Patients with LSTV showed significantly reduced motion between the transitional segment and the segment L5/S1 (RoM L5/S1) compared to the control group looking at the movement from disc deformation measured as the segmental wedge angle (p<0.001). This reduced segmental RoM resulted for segmental wedge angle from significantly reduced ventral flexion (p = 0.007) as well as dorsal extension (p = 0.001).

Looking at the segmental lordosis angle a reduced RoM in the transitional segment compared to control group was also detected (p = 0.035). For segmental lordosis angle the reduced RoM mainly resulted of significantly reduced dorsal extension (p = 0.043) whereas ventral flexion (p = 0.068) presented insignificant differences.

**Fig 3:**
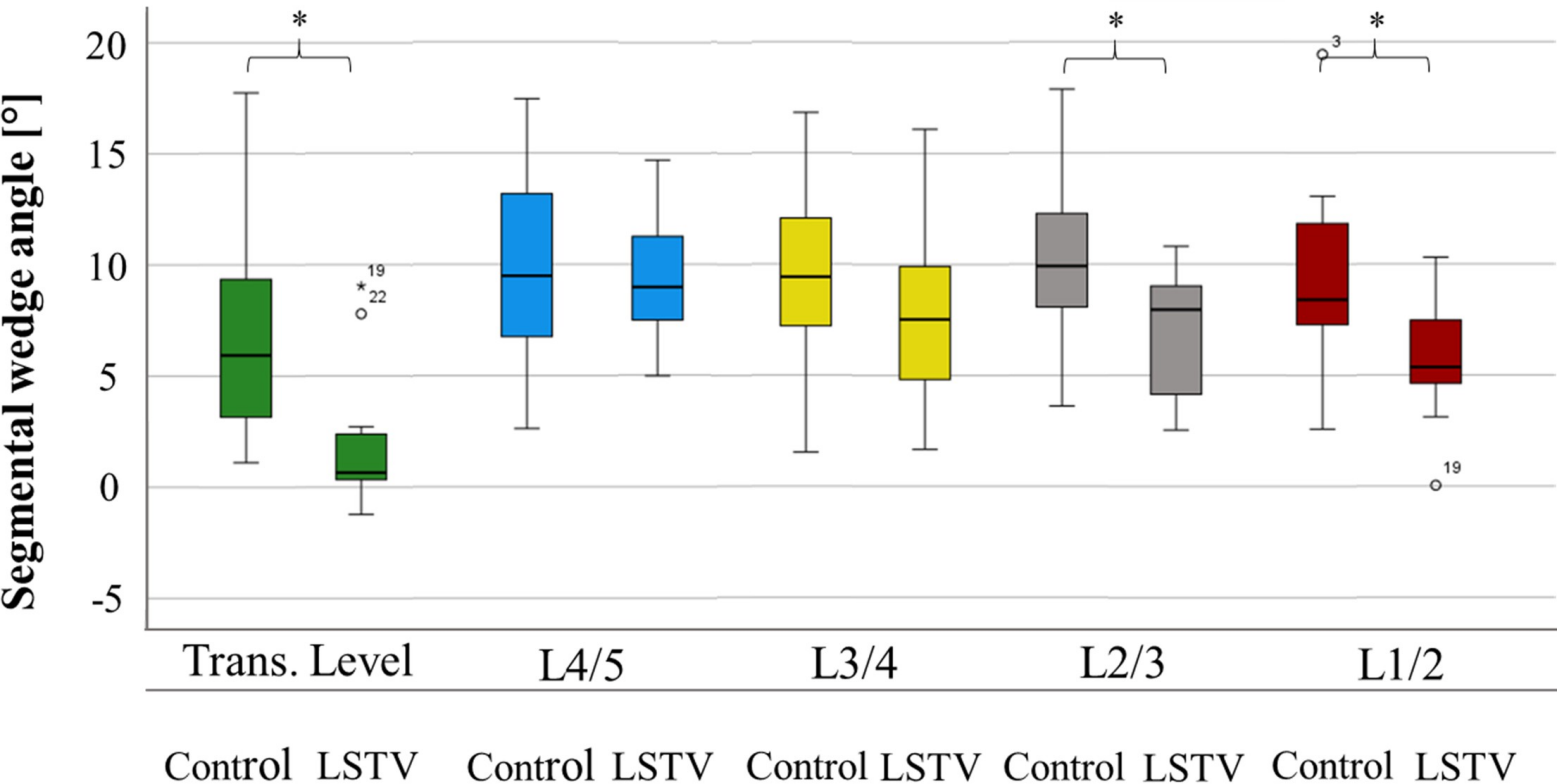
Segmental range of motion. Fig 3 compares the segmental range of motion based on the difference between segmental wedge angles in maximum ventral flexion and dorsal extension (Δ) of patients with LSTV and the control group. In the transitional segment, the range of motion between patients with LSTV and the control group differs significantly as well as in the L1/2 and L2/3 segment. Trans. Level = transitional vertebra level or level L5/S1 in patients without LSTV, Control = control group, LSTV = patient group with lumbosacral transitional vertebrae. The significance level was set at 0.05. Significant values are marked with an asterisk.

In the two cranial segments adjacent to the transitional segment, patients with LSTV did not differ significantly in their range of motion from the control group in segmental wedge angle (L4/5 p = 0.666, L3/4 p = 0.210) as well as in segmental lordosis angle (L4/5 p = 0.117, L3/4 p = 0.096). In the two most cranial lumbar segments, patients with LSTV showed significantly reduced range of motion for both segmental wedge angle (L2/3 p = 0.025, L1/2 p = 0.015) and segmental lordosis angle (L2/3 p = 0.028, L1/2 p = 0.004). The segmental mobility is shown in Table 3, segmental range of motion is presented in Fig 3.

**Table 3:** Segmental lumbar lordosis range of motion in patients with LSTV and control group.

	Segmental wedge angle			Segmental lordosis angle		
	LSTV mean (SD)	Control mean (SD)	p-value	LSTV mean (SD)	Control mean (SD)	p-value
Flex. Trans. Seg./ L5/S1 [°]	0.8 (±2.0)	3.2 (±3.3)	**0.007**	2.3 (±3.5)	5.0 (±5.4)	0.068
Ext. Trans. Seg./ L5/S1 [°]	0.8 (±1.0)	4.0 (±2.9)	**0.001**	2.2 (±2.2)	4.1 (±2.8)	**0.043**
RoM Trans. Seg./ L5/S1 [°]	1.7 (±1.9)	7.2 (±5.1)	**<0.001**	4.5 (±5.0)	9.1 (±6.4)	**0.035**
Flex. L4/5 [°]	6.1 (±3.4)	7.4 (±4.1)	0.382	8.8 (±5.5)	10.7 (±5.5)	0.316
Ext. L4/5 [°]	3.3 (±2.1)	2.7 (±2.1)	0.530	3.1 (±2.7)	4.3 (±3.3)	0.243
RoM L4/5 [°]	9.4 (±2.7)	10.1 (±4.6)	0.666	11.9 (±4.8)	15.0 (±5.7)	0.117
Flex. L3/4 [°]	5.0 (±4.1)	6.9 (±4.0)	0.210	7.1 (±3.9)	9.9 (±6.0)	0.162
Ext. L3/4 [°]	2.8 (±2.5)	2.6 (±1.7)	0.981	1.8 (±2.4)	2.2 (±3.4)	0.660
RoM L3/4 [°]	7.7 (±4.1)	9.5 (±4.2)	0.210	8.9 (±3.3)	12.1 (±6.7)	0.096
Flex. L2/3 [°]	4.2 (±3.8)	6.9 (±4.3)	**0.047**	6.1 (±3.1)	8.3 (±4.6)	0.081
Ext. L2/3 [°]	2.7 (±2.3)	2.9 (±2.3)	0.801	2.6 (±2.8)	3.3 (±2.1)	0.309
RoM L2/3 [°]	6.9 (±2.8)	9.9 (±3.5)	**0.025**	8.7 (±3.6)	11.6 (±4.1)	**0.035**
Flex. L1/2 [°]	3.1 (±2.6)	6.1 (±3.6)	**0.015**	4.7 (±3.0)	6.4 (±3.4)	0.177
Ext. L1/2 [°]	2.8 (±2.0)	3.2 (±2.4)	0.643	2.6 (±3.2)	5.4 (±2.2)	**0.006**
RoM L1/2 [°]	5.9 (±2.6)	9.3 (±4.2)	**0.015**	7.3 (±3.6)	11.7 (±4.6)	**0.004**

Table 3 gives the segmental movement in flexion and extension with the segmental wedge angles and the segmental lordosis angles of the lumbar spine. In the segment L5/S1-LSTV the motion of the transitional segment or in the absence of LSTV the motion of the segment L5/S1 was shown. The range of motion of each segment was determined based on the sum of the flexion and extension. LSTV = lumbo-sacral transitional vertebra, SD = standard deviation, RoM = range of motion, Flex. = ventral flexion, Ext. = dorsal extension. Level of significance was set at 0.05. Significant values are marked in bold.

### Distribution of lumbar mobility

Patients with LSTV differed significantly from the control group in the distribution of segmental mobility. While in patients without LSTV 16.2% of the total lumbar RoM–measured as a sum of all segmental wedge angles occurred in the segment L5/S1, in patients with LSTV 5.7% of the lumbar mobility resulted from the transitional segment (p = 0.002) as shown in Table 4. In contrast, in patients with LSTV 30.7% of lumbar motility resulted from the cranial adjacent segment to the LSTV, whereas in the control group, 21.6% of the lumbar flexibility resulted from the segment L4/5 (p = 0.007). In the upper lumbar segments L1/2 (p = 0.943), L2/3 (p = 0.723) and L3/4 (p = 0.266) patients with LSTV and the control group did not differ significantly in distribution of lumbar mobility.

**Table 4:** Distribution of lumbar mobility to lumbar segments.

	LSTV	Control group	p-value
Transitional Segment/ L5/S1	5.7%	16.2%	**0.002**
Cranial adjacent segment/ L4/5	30.7%	21.6%	**0.007**
L3/4	23.4%	20.4%	0.266
L2/3	21.4%	21.7%	0.723
L1/2	18.7%	20.1%	0.943

Table 4 shows the relative segmental lordosis based on the segmental wedge angle. Patients with LSTV have significantly reduced proportion of lumbar flexibility in the transitional segment and increased mobility in the cranial adjacent segment compared to the control group, whereas no differences was detected for the other lumbar segments.

### Influence of Castellvi grading of LSTV on segmental mobility

Expression and grading of LSTV, classified according to Castellvi, did not correlate significantly with a reduced absolute mobility in the transitional segment (p = 0.862, r = -0.046) nor with extended motion in the cranial adjacent segment (p = 0.674, r = -0.110).

## Discussion

This is the first study to investigate the segmental distribution of lumbar mobility in vivo in patients with LSTV with flexion-extension radiographs. Our results show that LSTV significantly alter the distribution of motion in the lumbar spine in vivo. While movement in the transitional segment was significantly reduced in patients with LSTV compared to segment L5/S1 of the control group, there was a significantly increased distribution of movement to the cranially adjacent segment.

In our cohort, 33.3% of patients presented with LSTV. The prevalence of LSTV in our study was therefore within the range of 5–36% prevalence reported in the literature [16–18]. The reported prevalence of LSTV differs, pending on the selected patients collective and to be dependent on regional factors [2, 12, 19–21]. Dzupa et al. reported a comparable prevalence of 27.6% in their study of a caucasian patients collective based on pelvic x-rays, unconnected to back pain [19]. While Hanhivaara et al. detected a prevalence of 21.1% in a Swedish collective of patients with back pain [12]. Haffer et al. reported a prevalence of 6.5% in a collective of patients without back pain from a central european collective [20]. Whereas Tang et al detected a prevalence of 15.8% in a Chinese Han population without pre-selection for back pain [2]. The study of Sekharappa et al. indicates the association between back pain and the presence of LSTV. In a study of an urological collective without back pain, he detected a prevalence of 8.1% whereas he reported a prevalence of 14% in a spine outpatient care department of the same hospital [21].

Patients with LSTV differed not significantly in lumbar lordosis compared to the control collective. There is no consensus in the literature regarding the effect of LSTV on lumbar lordosis [22–24]. While Chalian et al. and Mahato reported increased lumbar lordosis in patients with LSTV [22, 23], Abbas et al. reported no significant change in lumbar lordosis [24]. In our study, patients with LSTV showed a tendency towards a reduced lumbar range of motion compared with the control group, with statistical effects not reaching statistical significance. Whereas the summed segmental wedge angle showed significantly reduced lumbar flexion and lumbar RoM. These differences between the two measurement methods could result from the relatively small collective size and few patients with high-grade LSTV.

While lumbar flexion and lumbar RoM measured from the L1-S1 upper endplates did not differ significantly between patients with LSTV and the control group, significant differences were seen in the cumulated segmental wedge angle. Differences between the motion generated by intervertebral discs deformation and the motion of the completely lumbar spine, which includes a slight bony deformation of the vertebrae, have already been described by Been et al. [25]. Consequently, increased bony deformation may have occurred throughout the lumbar spine in patients with LSTV, compensating for decreased RoM as well as flexion by the intervertebral discs.

Looking into segmental movement, patients with LSTV had significantly reduced mobility of the transitional segment compared to segment L5/S1 of the control group, which is in line with the results of the cadaveric-study of Golubovsky et al. [13]. However, this investigation found a reduced range of motion in patients with LSTV, especially for axial torsion and side bending in vitro, but not for flexion or extension [13]. These differences may result from a tissue alteration in the cadaveric study of Golubovsky et al., the analysis of asymmetric LSTV only, and an isolated view on the osteo-ligamentous structures without inclusion of the musculature in their study. At the same time, the literature reports significant muscular adaptions in patients with LSTV [26, 27]. Recent evidence also indicates significant differences for the spinopelvic anatomy between LSTV and a control group, which might also affect the mutual interaction between the pelvis and lumbar spine [20].

No increased absolute range of motion compared to patients without LSTV was detected in the cranial adjacent segment to the transitional segment in patients with LSTV. Likewise, no significant differences in the range of motion of the segment L3/4 were seen. This is consistent with the findings of Golubovsky et al. [13]. In the upper segments of the lumbar spine L1/2 and L2/3, however, patients with LSTV showed a significantly reduced range of motion compared to the control group. Lee et al. reported a significant increased range of motion in the upper lumbar spine in the presence of degeneration of lower lumbar segment [28]. Accordingly, the differences in range of motion in the upper lumbar spine may result from compensatory increased motion in the upper lumbar spine due to the presence of osteochondrosis in the L5/S1 segment whereas in patients with LSTV this compensatory mechanism is possibly not sufficient due to the altered soft-tissue and osseous anatomy and accompanying changes in mobility of the transitional and adjacent segment.

Considering the relative proportions of the individual segments in the total lumbar motion, patients with LSTV and the control group showed significant differences. In accordance with the literature, the control group as well as patients with LSTV had the lowest mobility in the transitional segment and segment L5/S1 [29]. In both groups, this effect may have been further enhanced by the presence of osteochondrosis in segment L5/S1, as the degeneration could lead to a reduced range of motion [28]. However, patients with LSTV had significantly decreased mobility with a motion distribution in this segment of only 6.8% of total lumbar range of motion compared with 17.3% in the control group. Significant differences between patients with LSTV and the control group were also demonstrated in the cranial adjacent segment compared to the L4/5 segment. In patients with LSTV, 29.0% of the lumbar range of motion derived from the segment L4/5, whereas in the control group only 22.3% derived from this segment. In the upper lumbar spine, no differences in the distribution of the lumbar range of motion were observed between patients with LSTV and the control group. These changes in the distribution of the lumbar motion with a relative hypermobility of the cranial adjacent segment may be attributed to two causes, the anatomical variance with a weak iliolumbar ligament [10], and to a compensatory increased relative mobility due to the decreased mobility of the LSTV. Despite significantly higher distribution of the lumbar motion in the cranial adjacent segment, no differences in absolute range of motion were observed compared with segment L4/5 of the control group. This effect is might be influenced by the in tendency decreased overall lumbar range of motion in LSTV patients. No significant correlation between LSTV grading according to Castellvi and motion in the transitional segment or the cranial adjacent segment could be detected. However, reduced motion in the transitional segment and increased motion in the cranial adjacent segment would be expected due to the increased osseous connectivity of transversal process with sacral ala in higher Castellvi grading. These effects may could be not detected in our study due to the sample sizes with higher Castellvi gradings as well as the osteochondrosis in the transitional segment may could have diminished these expected effects.

Besides presenting the first analysis of the mobility of the lumbar spine in patients with LSTV in flexion-extension radiographs, the relatively small number of patients included need to be stated as a limitation of the study. The degree of degeneration of the lumbar spine was not compared between the groups, which may could have affected lumbar mobility. Apart from ventral flexion and dorsal extension, LSTV may also influence side-bending and rotation, which were not included in the analysis due to the retrospective study design and the lack of side-bending radiographs [30]. Detection of patients with LSTV in this study was performed using plain anterior posterior radiographs. However, the method with the highest sensitivity for the detection of LSTV is computed tomography or Ferguson radiographs [31].

## Conclusion

This is the first study to demonstrate that LSTV have a significant effect on lumbar spinal motion patterns with the use of flexion-extension radiographs. Patients with LSTV have reduced range of motion in the transitional segment. This is reflected in a reduced proportion to lumbar motion of only 6.8%. Consecutively, patients with LSTV have a significantly increased proportion of 29% of total lumbar motion in the cranial adjacent segment to the transitional segment. Thus, the reduced motion in the transitional segment as well as the increased proportion of mobility in the cranial adjacent segment can be considered as influencing factors for increased degeneration rates in the cranial adjacent segment to LSTV.

## Supporting information

S1 Dataset Minimal-data set of Fig 3 and Tables 2–4. (DOCX)
