# Supplementary material for: Lumbosacral transitional vertebrae alter the distribution of lumbar mobility–Preliminary results of a radiographic evaluation
Source: PLoS One. 2022 Sep 29;17(9):e0274581. doi: 10.1371/journal.pone.0274581 (PMC9521836; doi:10.1371/journal.pone.0274581)
Supplement: S1 Dataset — (DOCX) [file pone.0274581.s001.docx]

Minimal Data Set

| Fig # | Mean | S.D. | Statistical method | p-value | # samples |
| --- | --- | --- | --- | --- | --- |
| **Fig 3.** | | | | | |
| Trans. Level LSTV [°] | 1.7 | 1.9 | Students paired T-Test | **<0.001** | 17 |
| L5/S1 Control [°] | 7.2 | 5.1 |  |  | 17 |
| L4/5 LSTV [°] | 9.4 | 2.7 | Students paired T-Test | 0.666 | 17 |
| L4/5 Control [°] | 10.1 | 4.6 |  |  | 17 |
| L3/4 LSTV [°] | 7.7 | 4.1 | Students paired T-Test | 0.210 | 17 |
| L3/4 Control [°] | 9.5 | 4.2 |  |  | 17 |
| L2/3 LSTV [°] | 6.9 | 2.8 | Wilcoxon-rank sum Test | **0.025** | 17 |
| L2/3 Control [°] | 9.9 | 3.5 |  |  | 17 |
| L1/2 LSTV [°] | 5.9 | 2.6 | Students paired T-Test | **0.015** | 17 |
| L1/2 Control [°] | 9.3 | 4.2 |  |  | 17 |

| Table # | Mean | | | S.D. | | | Statistical method | | | p-value | | | # samples | | |
| --- | --- | --- | --- | --- | --- | --- | --- | --- | --- | --- | --- | --- | --- | --- | --- |
| **Table 2** | | | | | | | | | | | | | | | |
| S1-endplate to L1 upper endplate | | | | | | | | | | | | | | | |
| Lumbar lordosis LSTV [°] | 43.7 | | | 7.2 | | | Wilcoxon-rank sum Test | | | 0.875 | | | 17 | | |
| Lumbar lordosis Control [°] | 43.8 | | | 11.6 | | |  |  |  |  |  |  | 17 | | |
| RoM flexion Control [°] | 32.0 | | | 16.5 | | | Students paired T-Test | | | 0.083 | | | 17 | | |
| RoM flexion LSTV [°] | 44.1 | | | 19.2 | | |  |  |  |  |  |  | 17 | | |
| RoM extension LSTV [°] | 5.3 | | | 8.2 | | | Students paired T-Test | | | 0.426 | | | 17 | | |
| RoM extension Control [°] | 8.0 | | | 8.6 | | |  |  |  |  |  |  | 17 | | |
| Lumbar RoM LSTV [°] | 37.3 | | | 19.2 | | | Students paired T-Test | | | 0.065 | | | 17 | | |
| Lumbar RoM Control [°] | 52.1 | | | 20.5 | | |  |  |  |  |  |  | 17 | | |
| Segmental wedge angle | | | | | | | | | | | | | | | |
| Lumbar lordosis LSTV [°] | | 43.7 | | | 7.2 | | | Wilcoxon-rank sum Test | | | 0.875 | | | 17 | |
| Lumbar lordosis Control [°] | | 43.8 | | | 11.6 | | |  |  |  |  |  |  | 17 | |
| RoM flexion Control [°] | | 20.5 | | | 9.4 | | | Students paired T-Test | | | **0.017** | | | 17 | |
| RoM flexion LSTV [°] | | 30.7 | | | 13.5 | | |  |  |  |  |  |  | 17 | |
| RoM extension LSTV [°] | | 13.4 | | | 5.0 | | | Students paired T-Test | | | 0.535 | | | 17 | |
| RoM extension Control [°] | | 15.0 | | | 6.8 | | |  |  |  |  |  |  | 17 | |
| Lumbar RoM LSTV [°] | | 33.9 | | | 11.0 | | | Wilcoxon-rank sum Test | | | **0.022** | | | 17 | |
| Lumbar RoM Control [°] | | 45.8 | | | 14.8 | | |  |  |  |  |  |  | 17 | |
| **Table 3** | | | | | | | | | | | | | | | |
| Segmental wedge angle | | | | | | | | | | | | | | | |
| Flex. Trans. Seg. LSTV [°] | | | 0.8 | | | 2.0 | | | Wilcoxon-rank sum Test | | | **0.007** | | | 17 |
| Flex. L5/S1 Control [°] | | | 3.2 | | | 3.3 | | |  |  |  |  |  |  | 17 |
| Ext. Trans. Seg. LSTV [°] | | | 0.8 | | | 1.0 | | | Wilcoxon-rank sum Test | | | **0.001** | | | 17 |
| Ext. L5/S1 Control [°] | | | 4.0 | | | 2.9 | | |  |  |  |  |  |  | 17 |
| RoM. Trans. Seg. LSTV [°] | | | 1.7 | | | 1.9 | | | Students paired T-Test | | | **<0.001** | | | 17 |
| RoM. L5/S1 Control [°] | | | 7.2 | | | 5.1 | | |  |  |  |  |  |  | 17 |
| Flex. L4/5 LSTV [°] | | | 6.1 | | | 3.4 | | | Students paired T-Test | | | 0.382 | | | 17 |
| Flex. L4/5 Control [°] | | | 7.4 | | | 4.1 | | |  |  |  |  |  |  | 17 |
| Ext. L4/5 LSTV [°] | | | 3.3 | | | 2.1 | | | Students paired T-Test | | | 0.530 | | | 17 |
| Ext. L4/5 Control [°] | | | 2.7 | | | 2.1 | | |  |  |  |  |  |  | 17 |
| RoM. L4/5 LSTV [°] | | | 9.4 | | | 2.7 | | | Students paired T-Test | | | 0.666 | | | 17 |
| RoM. L4/5 Control [°] | | | 10.1 | | | 4.6 | | |  |  |  |  |  |  | 17 |
| Flex. L3/4 LSTV [°] | | | 5.0 | | | 4.1 | | | Students paired T-Test | | | 0.210 | | | 17 |
| Flex. L3/4 Control [°] | | | 6.9 | | | 4.0 | | |  |  |  |  |  |  | 17 |
| Ext. L3/4 LSTV [°] | | | 2.8 | | | 2.5 | | | Wilcoxon-rank sum Test | | | 0.981 | | | 17 |
| Ext. L3/4 Control [°] | | | 2.6 | | | 1.7 | | |  |  |  |  |  |  | 17 |
| RoM. L3/4 LSTV [°] | | | 7.7 | | | 4.1 | | | Students paired T-Test | | | 0.210 | | | 17 |
| RoM. L3/4 Control [°] | | | 9.5 | | | 4.2 | | |  |  |  |  |  |  | 17 |
| Flex. L2/3 LSTV [°] | | | 4.2 | | | 3.8 | | | Students paired T-Test | | | **0.047** | | | 17 |
| Flex. L2/3 Control [°] | | | 6.9 | | | 4.3 | | |  |  |  |  |  |  | 17 |
| Ext. L2/3 LSTV [°] | | | 2.7 | | | 2.3 | | | Students paired T-Test | | | 0.801 | | | 17 |
| Ext. L2/3 Control [°] | | | 2.9 | | | 2.3 | | |  |  |  |  |  |  | 17 |
| RoM. L2/3 LSTV [°] | | | 6.9 | | | 2.8 | | | Wilcoxon-rank sum Test | | | **0.025** | | | 17 |
| RoM. L2/3 Control [°] | | | 9.9 | | | 3.5 | | |  |  |  |  |  |  | 17 |
| Flex. L1/2 LSTV [°] | | | 3.1 | | | 2.6 | | | Students paired T-Test | | | **0.015** | | | 17 |
| Flex. L1/2 Control [°] | | | 6.1 | | | 3.6 | | |  |  |  |  |  |  | 17 |
| Ext. L1/2 LSTV [°] | | | 2.8 | | | 2.0 | | | Students paired T-Test | | | 0.643 | | | 17 |
| Ext. L1/2 Control [°] | | | 3.2 | | | 2.4 | | |  |  |  |  |  |  | 17 |
| RoM. L1/2 LSTV [°] | | | 5.9 | | | 2.6 | | | Students paired T-Test | | | **0.015** | | | 17 |
| RoM. L1/2 Control [°] | | | 9.3 | | | 4.2 | | |  |  |  |  |  |  | 17 |
| Segmental lordosis angle | | | | | | | | | | | | | | | |
| Flex. Trans. Seg. LSTV [°] | | | 2.3 | | | 3.5 | | | Wilcoxon-rank sum Test | | | 0.068 | | | 17 |
| Flex. L5/S1 Control [°] | | | 5.0 | | | 5.4 | | |  |  |  |  |  |  | 17 |
| Ext. Trans. Seg. LSTV [°] | | | 2.2 | | | 2.2 | | | Students paired T-Test | | | **0.043** | | | 17 |
| Ext. L5/S1 Control [°] | | | 4.1 | | | 2.8 | | |  |  |  |  |  |  | 17 |
| RoM. Trans. Seg. LSTV [°] | | | 4.5 | | | 5.0 | | | Wilcoxon-rank sum Test | | | **0.035** | | | 17 |
| RoM. L5/S1 Control [°] | | | 9.1 | | | 6.4 | | |  |  |  |  |  |  | 17 |
| Flex. L4/5 LSTV [°] | | | 8.8 | | | 5.5 | | | Students paired T-Test | | | 0.316 | | | 17 |
| Flex. L4/5 Control [°] | | | 10.7 | | | 5.5 | | |  |  |  |  |  |  | 17 |
| Ext. L4/5 LSTV [°] | | | 3.1 | | | 2.7 | | | Wilcoxon-rank sum Test | | | 0.243 | | | 17 |
| Ext. L4/5 Control [°] | | | 4.3 | | | 3.3 | | |  |  |  |  |  |  | 17 |
| RoM. L4/5 LSTV [°] | | | 11.9 | | | 4.8 | | | Students paired T-Test | | | 0.117 | | | 17 |
| RoM. L4/5 Control [°] | | | 15.0 | | | 5.7 | | |  |  |  |  |  |  | 17 |
| Flex. L3/4 LSTV [°] | | | 7.1 | | | 3.9 | | | Students paired T-Test | | | 0.162 | | | 17 |
| Flex. L3/4 Control [°] | | | 9.9 | | | 6.0 | | |  |  |  |  |  |  | 17 |
| Ext. L3/4 LSTV [°] | | | 1.8 | | | 2.4 | | | Students paired T-Test | | | 0.660 | | | 17 |
| Ext. L3/4 Control [°] | | | 2.2 | | | 3.4 | | |  |  |  |  |  |  | 17 |
| RoM. L3/4 LSTV [°] | | | 8.9 | | | 3.3 | | | Students paired T-Test | | | 0.096 | | | 17 |
| RoM. L3/4 Control [°] | | | 12.1 | | | 6.7 | | |  |  |  |  |  |  | 17 |
| Flex. L2/3 LSTV [°] | | | 6.1 | | | 3.1 | | | Students paired T-Test | | | 0.081 | | | 17 |
| Flex. L2/3 Control [°] | | | 8.3 | | | 4.6 | | |  |  |  |  |  |  | 17 |
| Ext. L2/3 LSTV [°] | | | 2.6 | | | 2.8 | | | Wilcoxon-rank sum Test | | | 0.309 | | | 17 |
| Ext. L2/3 Control [°] | | | 3.3 | | | 2.1 | | |  |  |  |  |  |  | 17 |
| RoM. L2/3 LSTV [°] | | | 8.7 | | | 3.6 | | | Wilcoxon-rank sum Test | | | **0.035** | | | 17 |
| RoM. L2/3 Control [°] | | | 11.6 | | | 4.1 | | |  |  |  |  |  |  | 17 |
| Flex. L1/2 LSTV [°] | | | 4.7 | | | 3.0 | | | Wilcoxon-rank sum Test | | | 0.177 | | | 17 |
| Flex. L1/2 Control [°] | | | 6.4 | | | 3.4 | | |  |  |  |  |  |  | 17 |
| Ext. L1/2 LSTV [°] | | | 2.6 | | | 3.2 | | | Wilcoxon-rank sum Test | | | **0.006** | | | 17 |
| Ext. L1/2 Control [°] | | | 5.4 | | | 2.2 | | |  |  |  |  |  |  | 17 |
| RoM. L1/2 LSTV [°] | | | 7.3 | | | 3.6 | | | Students paired T-Test | | | **0.004** | | | 17 |
| RoM. L1/2 Control [°] | | | 11.7 | | | 4.6 | | |  |  |  |  |  |  | 17 |

| Table # | Mean [°] | S.D. [°] | % | Statistical method | p-value | # samples |
| --- | --- | --- | --- | --- | --- | --- |
| **Table 4** | | | | | | |
| Transitional Segment LSTV | 1.7 | 1.9 | 5.7 | Wilcoxon-rank sum Test | **0.002** | 17 |
| L5/S1 Control | 7.2 | 5.1 | 16.2 |  |  | 17 |
| Cranial adjacent segment/ L4/5 LSTV | 9.4 | 2.7 | 30.7 | Wilcoxon-rank sum Test | **0.007** | 17 |
| Cranial adjacent segment/ L4/5 Control | 10.1 | 4.6 | 21.6 |  |  | 17 |
| L3/4 LSTV | 7.7 | 4.1 | 23.4 | Wilcoxon-rank sum Test | 0.266 | 17 |
| L3/4 Control | 9.5 | 4.2 | 20.4 |  |  | 17 |
| L2/3 LSTV | 6.9 | 2.8 | 21.4 | Wilcoxon-rank sum Test | 0.723 | 17 |
| L2/3 Control | 9.9 | 3.5 | 21.7 |  |  | 17 |
| L1/2 LSTV | 5.9 | 2.6 | 18.7 | Wilcoxon-rank sum Test | 0.943 | 17 |
| L1/2 Control | 9.3 | 4.2 | 20.1 |  |  | 17 |
